# Fabrication of Titanium-Niobium-Zirconium-Tantalium Alloy (TNZT) Bioimplant Components with Controllable Porosity by Spark Plasma Sintering

**DOI:** 10.3390/ma11020181

**Published:** 2018-01-24

**Authors:** Jack Rechtin, Elisa Torresani, Eugene Ivanov, Eugene Olevsky

**Affiliations:** 1 Mechanical Engineering, San Diego State University, 5500 Campanile Dr., San Diego, CA 92182, USA; jrechtin@gmail.com (J.R.); etorresani@sdsu.edu (E.T.); 2 Tosoh SMD Inc., Grove City, OH 43123, USA; Eugene.Ivanov@tosoh.com; 3 NanoEngineering, University of California, 9500 Gilman Dr., San Diego, La Jolla, CA 92182, USA

**Keywords:** Spark Plasma Sintering, porosity, TNZT, bioimplant

## Abstract

Spark Plasma Sintering (SPS) is used to fabricate Titanium-Niobium-Zirconium-Tantalum alloy (TNZT) powder—based bioimplant components with controllable porosity. The developed densification maps show the effects of final SPS temperature, pressure, holding time, and initial particle size on final sample relative density. Correlations between the final sample density and mechanical properties of the fabricated TNZT components are also investigated and microstructural analysis of the processed material is conducted. A densification model is proposed and used to calculate the TNZT alloy creep activation energy. The obtained experimental data can be utilized for the optimized fabrication of TNZT components with specific microstructural and mechanical properties suitable for biomedical applications.

## 1. Introduction

One of the biggest challenges in orthopedic implant design is developing implants with comparable mechanical properties to the bone they interface with and replace [1,2,3,4]. It has been shown that a mismatch in bone and implant elastic moduli can lead to premature bone failure [5,6,7,8].

A variety of ceramic materials have been used as orthopedic implants depending on the given requirements of the specific implant. However, these come with possible problems. For instance, β-tricalcium phosphate (β-TCP) has been shown cause inflammation and giant cell reaction [9]. Another ceramic, hydroxyapitite, is often used as a coating for implants, but its wear debris can stimulate the release of cytokines, causing the implants to fail [10,11].

Certain polymers, such as polymethylmetacrylate (PMMA), have mechanical properties suitable for bone substitution and bone cement. While this material has been useful in application, it shows poor osteointegration and may inhibit bone healing due to monomer toxicity resulting from the heat treatment during the curing of PMMA [12,13]. The use of PMMA can also result in aseptic loosening due to an interface membrane that can form locally [14,15].

The most common metal used in orthopedic implants is titanium [16,17,18,19]. Titanium has shown itself to be acceptably biocompatible and is sufficiently strong to withstand loads seen at high stress locations such as the hip. However, even though titanium is substantially more flexible than ceramic material, it is still stiffer than bone. Therefore, a number of titanium alloys have been developed to obtain a lower elastic modulus while maintaining adequate strength. 

One of the most common metal alloys used for orthopedic implants is Ti-6Al-4V [7,17]. This alloy is less likely to corrode in the body than pure titanium, making it more biocompatible. Also, it has a lower stiffness than pure titanium, so it is also a better choice purely in terms of its mechanical properties. However, the stiffness of this alloy is still higher than that of bone, so more alloys have been developed and tested.

One of the most promising materials for future orthopedic implants is Titanium-Niobium-Zirconium-Tantalum alloy (TNZT) [17,20,21,22,23,24]. This high entropy titanium alloy has a lower elastic modulus than Ti-6Al-4V, and is even less likely to corrode in the body than Ti-6Al-4V. It is still strong enough to withstand typical loading seen by the bones it would implant, but the reduced stiffness helps mitigate the chances of delamination between the bone and the implant. 

In this connection, it may be useful for titanium alloy orthopedic implants to retain a certain level of porosity [25,26]. Bandyopadhyay et al. studied the influence of porosity on Ti-6Al-4V implants [7]. They found that the porosities resulting in the most similar elastic modulus to bone were in the range of 23–32%. 

The TNTZ alloy has a microstructure including a matrix of body centered cubic (BCC) β phase with hexagonal close packed (HCP) α phase grain (crystallite) boundary precipitates. The predominant amount of β phase is the cause of the low value of elastic modulus that this alloy shows.

It is important to understand the different methods that can be used to yield an acceptable final porosity. One way to produce porous components is to introduce a secondary material that can be removed from the sample during or after sintering [27]. The disadvantage of using a secondary material is the time and complexity of the process. 

The second strategy for creating porous components is partially sintering loose powders or fibers. If a low sintering temperature and low pressure are used during consolidation, residual porosity can be achieved. The main advantage to this method is the time, energy, and cost savings. Samples can be produced quickly, loose powder can be used directly without pre-compression or binding, and no intermediate materials are needed to create the pore structure.

SPS has been shown to achieve faster densification than traditional sintering techniques [28,29,30,31,32]. Therefore, by creating porous components using SPS, a maximum amount of time saving can be achieved. Not only can using SPS reduce the time it takes to produce each specimen, but it also allows the operator to quickly determine optimal sintering parameters.

## 2. Experimental

### 2.1. Powder Characterization

The material examined in this study is Ti-35 wt % Nb-7 wt % Zr-5 wt % Ta (TNZT) produced by Tosoh Co. (Tokyo, Japan). Two sets of powdered TNZT have been used. The first set had a nominal particle size of 150 μm and the second had a nominal particle size of 300 μm. Both of these powders were subjected to the same characterization, fabrication, and testing procedures, described below.

The theoretical density of the powdered TNZT material was measured by pycnometry analysis, using a Micromeritics AccuPyc 1330. The resulting density values were used to determine the relative density of the sintered samples.

The differential scanning calorimetry (DSC) analysis was carried out in a TA Instruments SDTQ600 using 100 mL/min of argon atmosphere, increasing the temperature from 40 °C to 1500 °C at a rate of 10 °C/min and cooled in air. 

Both the 150 μm and the 300 μm powders were subjected to SEM and EDS analysis using a Quanta 450 FEG.

### 2.2. SPS Process

All of the TNZT samples were spark plasma sintered using a 15 mm die. Graphite paper surrounded the samples to keep them from directly contacting the SPS tooling. Once the powder was contained in the die and surrounded with graphite paper, it was subjected to SPS using a Dr. Sinter Lab Spark Plasma Sintering System.

As discussed later, the sintering regimes covered temperatures from 400 °C to 800 °C, pressures from 20 MPa to 60 MPa, and holding times from 0 min to 15 min. All samples were sintered with roughly a 10 Pa level of vacuum and a pulse ratio of 12 ms on and 2 ms off. The temperature was increased at a rate of 100 °C/min, and the specimen thickness ranged from 1.5 mm to 3 mm depending on the relative density reached.

### 2.3. Microstructure and Mechanical Prorpietes Characterization

After the samples were removed from the SPS tooling, they were ground with SiC paper to remove any residual carbon that remained on the outer surface or in the surface pores of the samples. They were then sonicated in water for 10 min to remove any SiC particles that may have been acquired during grinding. The samples were then dried and the pertinent relative density measurements needed to use the Archimedes method were taken.

Electron Backscatter Diffraction (EBSD, FEI, Hillsboro, OR, USA) analysis was conducted to examine the crystalline orientation of the processed structure. 

The Vickers hardness (HV) of the samples was calculated using a Wilson Instruments 402MVD Knoop/Vickers hardness tester (Wilson Wolpert Instrument, Aachen, Germany). 

The samples were ground and polished with increasingly fine SiC abrasive paper. The samples were then sonicated in water for 10 min to remove any residual SiC that embedded itself in the samples. After this, the samples were dried and subjected to hardness testing.

The HV for each sample was calculated by indenting the sample with a 136° square-based right pyramid indenter for 10 s. A fixed load of 1 kN was used and the area of indentation was measured using optical encoders. The hardness was then calculated using the indentation force and area. Since this method measures micro-hardness, it was measured in multile locations for each sample. The obtained values were then averaged to determine the HV of each sample. 

The samples were cut in half using an Isomet 11-4245 diamond wafering blade (Buehler, Lake Bluff, IL, USA). Then, each sample was ground and polished with SiC grinding paper with increasing grit rating. Finally, each sample was sonicated in water for 10 min to remove any residual SiC particles that were acquired during the grinding and polishing processes. Once this was complete, the samples were dried and subjected to SEM (FEI, Hillsboro, OR, USA) analysis using the same microscope mentioned earlier in this report.

### 2.4. Densification Model

A model based on the continuum theory of sintering [29] is proposed in order to describe the densification of TNZT. For this purpose, the kinetic Equation (1) [30] is used. This is an example of an equation:

(1) $$\dot{\theta }=-A{\left(\frac{3\theta }{2}\right)}^{\frac{m+1}{2m}}{\left(1-\theta \right)}^{\frac{m-3}{2m}}{\left(\frac{\sigma_{z}}{G}\right)}^{\frac{1}{m}}$$

The above-mentioned equation relates the porosity evolution of the specimen (*ϴ*) with consolidation parameters *m* and *Q*, respectively, strain rate sensitivity and activation energy. The parameter *σz* is the external stress, *G* the shear modulus, and *A* can be expressed using Equation (2) [30]:

(2) $$A=\left[\frac{G}{A_{0}T}{\left(\frac{b}{d}\right)}^{p}exp\left(\frac{-Q}{RT}\right)\right]$$

where *b* is the Burgers vector, *d* is the grain size, *p* is the grain size parameter, and *T* is the sintering temperature. *A*_0_ is a parameter that depends on the material creep constant, diffusion coefficient, and Burgers vector. The values of the creep activation energy *Q* have been estimated using a regression analysis to obtain the best fit to the experimental data. The strain rate sensitivity value m was chosen based on the experimental data known in the literature for Ti (*m* = 0.23) [31].

## 3. Results

The pycnometry results showed that the theoretical densities of the 150 μm and 300 μm powders were 5.72 g/cm^3^ and 5.75 g/cm^3^, respectively. This slight variation in theoretical density could be a result of impurities in the powders.

**Figure 1:**
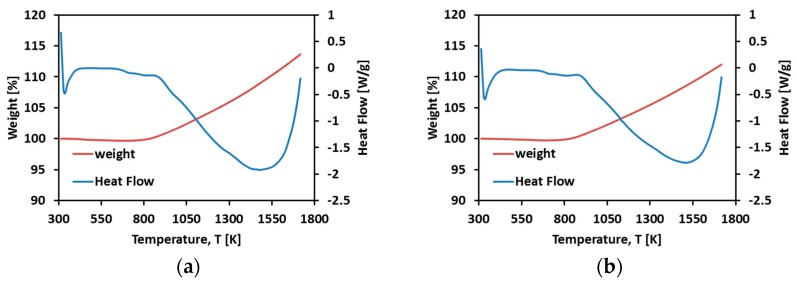
TGA-DSC curves of (**a**) 150 μm TNZT powder and (**b**) 300 μm TNZT powder.

The DSC and TGA analysis (Figure 1) showed that both powders were stable in the temperature range which the experiments were planned to fall within. The marked weight gain starting at 500 °C is due to the apparent effect of the oxidation (due to the protected environment of the SPS vacuum chamber no oxidation was possible during sample processing).

**Figure 2:**
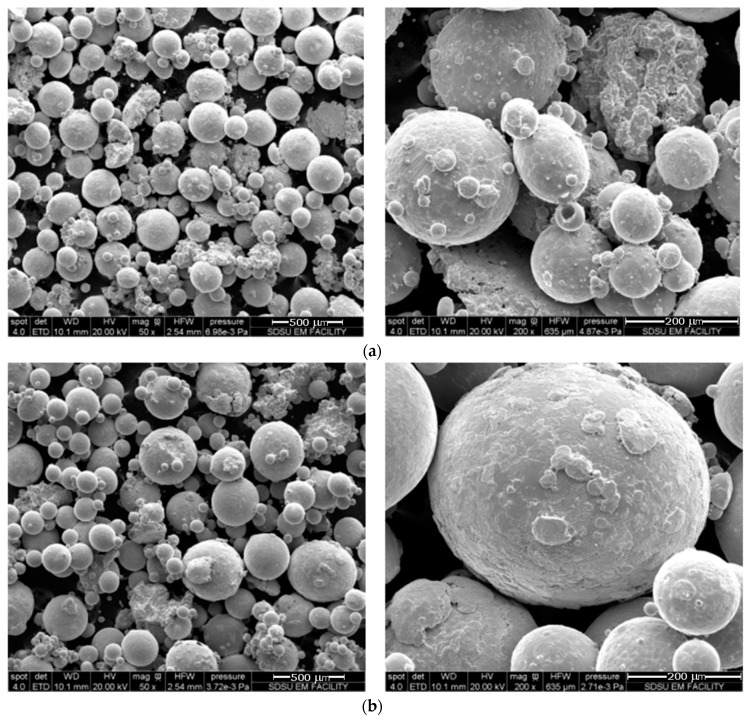
SEM images of (**a**) 150 μm TNZT powder (50× and 200×) and (**b**) 300 μm TNZT powder (50× and 200×).

Figure 2 shows the SEM images of both the 150 μm and 300 μm TNZT powder at different magnification levels.

The SEM analysis of both powders shows a wide range of particle sizes. The nominally 150 μm powder does show a smaller average particle size than the nominal average size of the 300 μm powder, but both powders contain particles as small as 40 um.

It is also interesting to note that both powders include non-spherical particles. The majority of particles in both powders are spherical, but some amount of an abnormal non-spherical particle morphology is present in both powders. EDS analysis was conducted on the spherical particles and the abnormal particles in each powder sample. The results of the EDS analysis on the spherical and irregular TNZT particles show that the relative amounts of Ti, Zr, Nb, and Ta are similar. This indicates that both particle morphologies correspond to the TNZT stoichiometry. 

**Figure 3:**
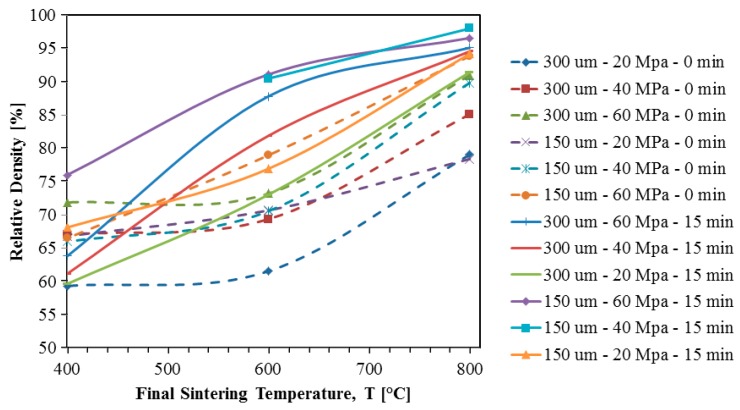
Relative density of all TNZT samples subjected to SPS with a heating rate of 100 °C/min, where the dashed line refers to the cases without the holding time, and the solid line with 15 min of holding time.

Figure 3 shows relative density curves for all samples to give the reader an understanding of the trends in final densification due to initial particle size, sintering pressure, final sintering temperature, and holding time. This figure thereby represents a “densification map”.

The effects of varying the final sintering temperature, pressure, holding time, and particle size on the final densification of samples has been the subject of many studies in the past. In the present study, these relationships are investigated specifically for TNZT samples produced using SPS. After consulting the literature, the following trends were anticipated:

❖ Increasing the sintering temperature should increase the final sample density [29];
❖ Increasing the sintering pressure should increase the final sample density [29];
❖ Increasing the holding time should increase the final sample density [32];
❖ Decreasing the initial particle size should increase the final sample density [33];
❖ Using designed particle distributions can be employed to tune the compaction density [31].

The solid lines in Figure 3 show that each powder for various holding times and pressure combinations experiences an increasing densification as temperature is increased. Also, the expected trend that smaller particles would densify more than larger particles when subjected to the same sintering conditions is shown in most cases. 

It is also clear from these data that when sintering at 400 °C, the initial particle size impacts the final densification more than the pressure. However, as temperature increases, this is not the case. At 600 °C, the trend shows that the pressure is a more dominant factor than the particle size for determining the final relative density. However, it should be noted that at each pressure, the samples with smaller initial particle sizes still reach higher relative densities.

At the highest temperature of 800 °C, the dependence of relative density on both the initial particle size and the sintering pressure becomes less apparent. This is the case because at 800 °C, all of the samples are nearly fully dense, so as long as 800 °C is reached and held for 15 min, it seems that the particle size and sintering pressure are inconsequential to the final relative density.

The difference between the final relative densities of samples sintered with and without a 15 min holding time is quite interesting. By examining Figure 3, it is clear that adding the 15 min holding time significantly increases the densification of most samples, with the exception of the samples that reach nearly full density without a holding time. 

The dashed lines in Figure 3 show that without the 15 min holding time, the increase in density when raising the final sintering temperature from 400 °C to 600 °C is very limited. The only set of samples that shows an increase of over 5% relative density in this range of temperatures is the 150 μm powder subjected to 60 MPa. This is the combination of particle size and pressure most conducive to sintering, so it makes sense that it would experience more rapid densification at lower temperatures than the other samples, but it is unexpected that so little densification occurs between the temperatures of 400 °C and 600 °C for the other samples. The most likely explanation of this phenomenon is that for low sintering pressures and large initial particle sizes, the samples are still in the initial stage of sintering when they reach 600 °C. Particles are beginning to coalesce and form necks in the initial stage of sintering, but densification is minimal. Significant densification does not begin until the intermediate stage of sintering, so it appears that the only sample to reach the intermediate stage of sintering at 600 °C is the 150 μm sample sintered under 60 MPa. This makes sense, as this sample has a small particle size and is subjected to the maximum pressure, making it the most sinterable sample.

Also, the trends regarding the impact of particle size and pressure on the final density of the samples are much less apparent when the holding time is removed. The one set of samples that seems to still follow these trends at each final sintering temperature is the 300 μm powder with a sintering pressure of 20 MPa, as it still results in the lowest final densities at each temperature. 

However, at 400 °C, the 300 μm powder pressed at 60 MPa ends up with a higher relative density than the 150 μm powder subjected to the same pressure. This goes against the trend seen when examining the solid lines in Figure 3, which showed that smaller particle sizes yielded greater densification at 400 °C. Additionally, five of the six samples sintered at 400 °C without holding time all show virtually identical relative densities so it is very difficult to tell how much of an effect the pressure and particle size really have on the final densities of the samples sintered at this temperature.

However, the same trends are followed for all samples at the higher temperatures. At 600 °C and 800 °C, the relative density of the samples increases as pressure is increased and particle size is reduced.

It is also worth noting that a final relative density greater that 90% was only reached in half of the samples. 

**Figure 4:**
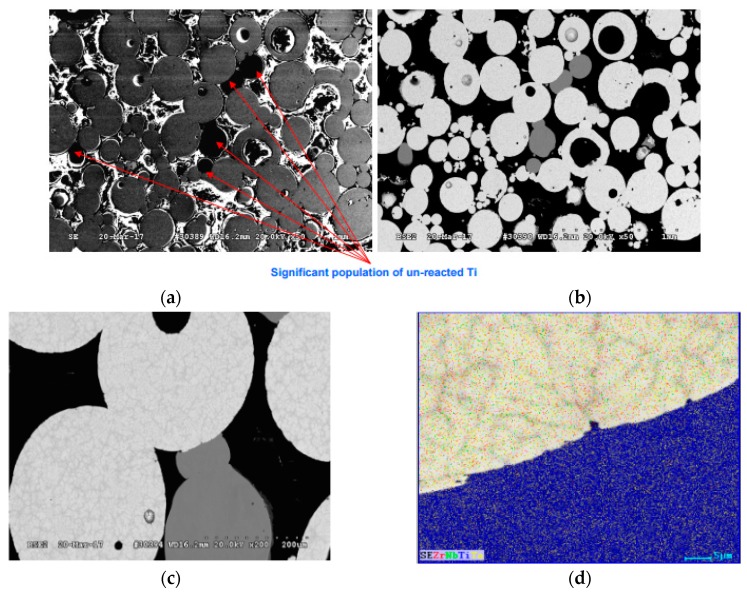
SEM (**a**-**c**) and EDS (**d**) analysis of TNZT sample sintered at 400 °C with 60 MPa and 15 min of holding time that shows 68% of relative density and contains unreacted titanium. In the map shown in (**d**), the Red refers to Zr, Green to Nb, Blue to Ti, and Yellow to Ta.

**Figure 5:**
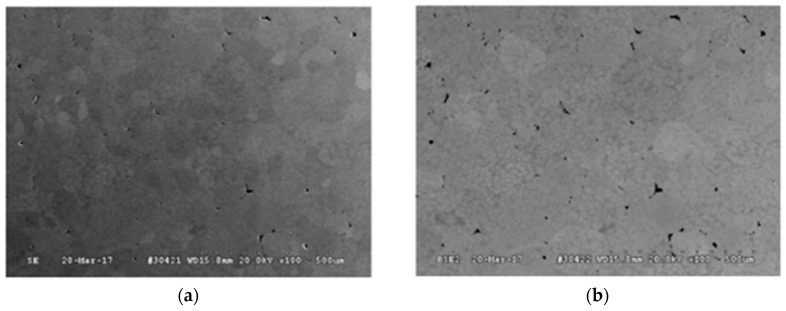

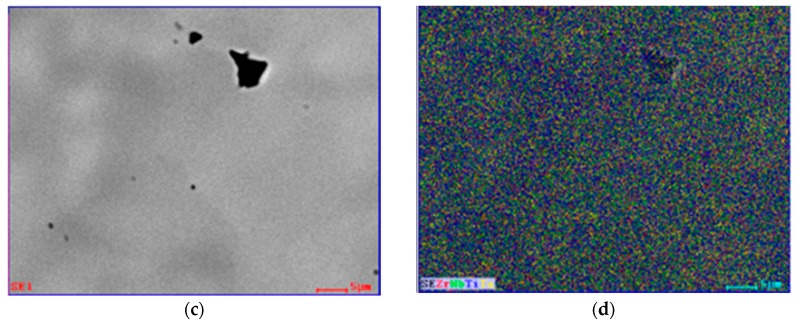
SEM (**a**–**c**) and EDS (**d**) analysis TNZT sample sintered at 600 °C with 60 MPa and 15 min of holding time that shows 85% of relative density and contains no unreacted titanium. In the map shown in (**d**), the Red refers to Zr, Green to Nb, Blue to Ti, and Yellow to Ta.

The EDS analysis indicated that some of the samples contained a significant amount of un-reacted titanium (Figure 4), but others did not (Figure 5).

The amount of reacted/unreacted titanium can be correlated with the SPS parameters as temperature, pressure, and holding time are shown to have an influence on promoting the diffusion of elements in the titanium.

**Figure 6:**
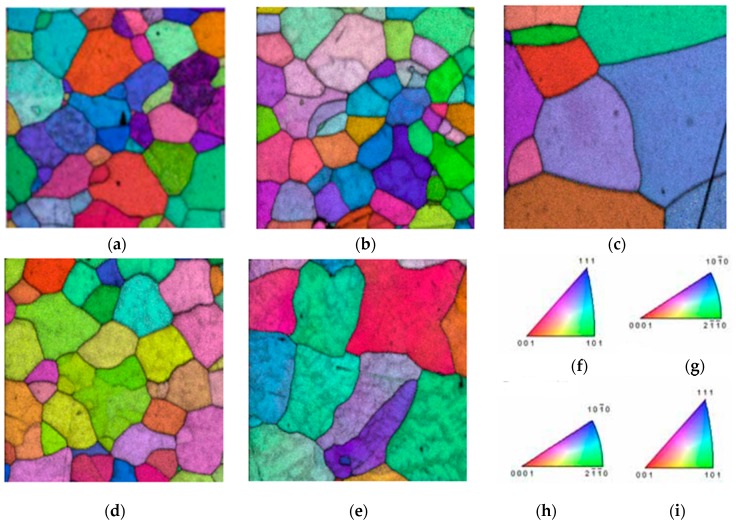
(**a**–**e**) EBSD inverse pole figure maps for five different samples and pole diagrams for (**f**) Tantalum; (**g**) Zirconium; (**h**) Titanium and (**i**) Niobium.

The grain orientation can also be assessed for selected samples (see Figure 6). 

The results show that there is no dominant direction for the grain lattice orientation, meaning that there is no imposed anisotropy on these samples due to their grain orientation. This is important because it also means that the macroscopic mechanical properties of the samples should be isotropic.

The Vickers hardness was collected for the samples sintered without holding time and the tensile strength was calculated from the Hardness Conversions for Steels in the Mechanical Testing and Evaluation section of the ASM handbook [34]. The TNZT samples with nearly full density have Vickers Hardness values near 160, which correlate to strengths of about 560 MPa using the ASM handbook. This is comparable to the strength of fully dense TNZT [35], so it has been determined that the ASM handbook conversion table gives an accurate approximation of TNZT strength.

**Figure 7:**
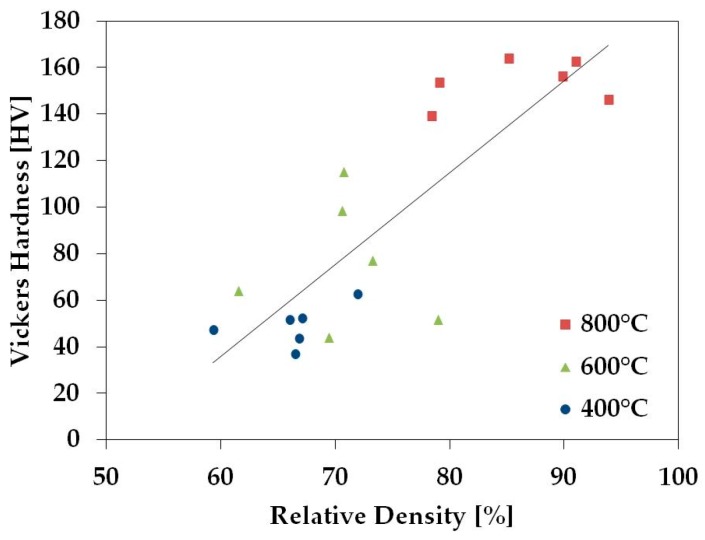
Average Vickers harness values for TNZT samples sintered at different temperatures (red square 800 °C, green triangle 600 °C, and blue circle 400 °C) without holding time and their relation to relative density.

**Figure 8:**
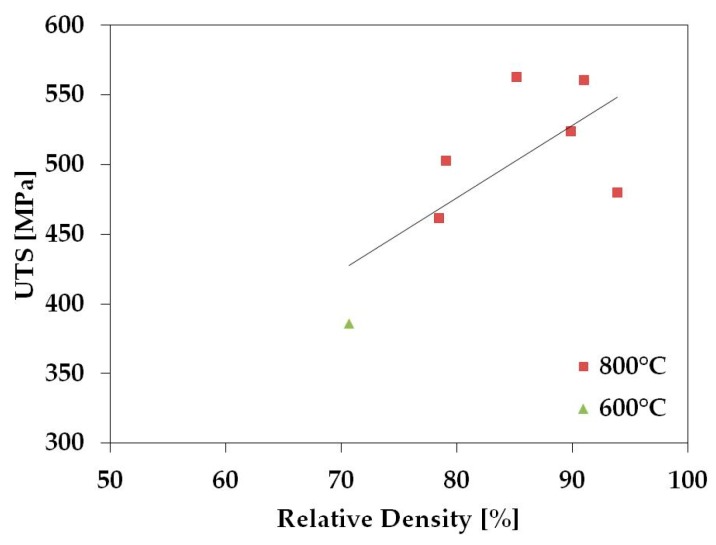
Ultimate tensile strength values for TNZT samples sintered at different temperatures (red square 800 °C and green triangle 600 °C) without holding time and their relation to relative density.

Figure 7 and Figure 8 show that the hardness and the strength of the TNZT samples tend to increase with relative density. This is expected, as this relationship has been shown in the literature for a variety of materials [36,37].

Although tensile tests to determine the elastic modulus of the sintered TNZT samples were not conducted, it is likely that comparable elastic moduli to that of cortical bone were achieved. As mentioned above, it has been shown that Ti-6Al-4V samples with 23–32% porosities have elastic moduli in the same range of cortical bone. Also, it has been shown that fully dense TNZT has a lower elastic modulus than fully dense Ti-6Al-4V and that increasing porosity reduces the elastic modulus of a material. 

In this study, TNZT samples with porosities of up to 40% were produced. Therefore, it should be expected that these samples have even lower elastic moduli than those discussed in [7].

**Figure 9:**
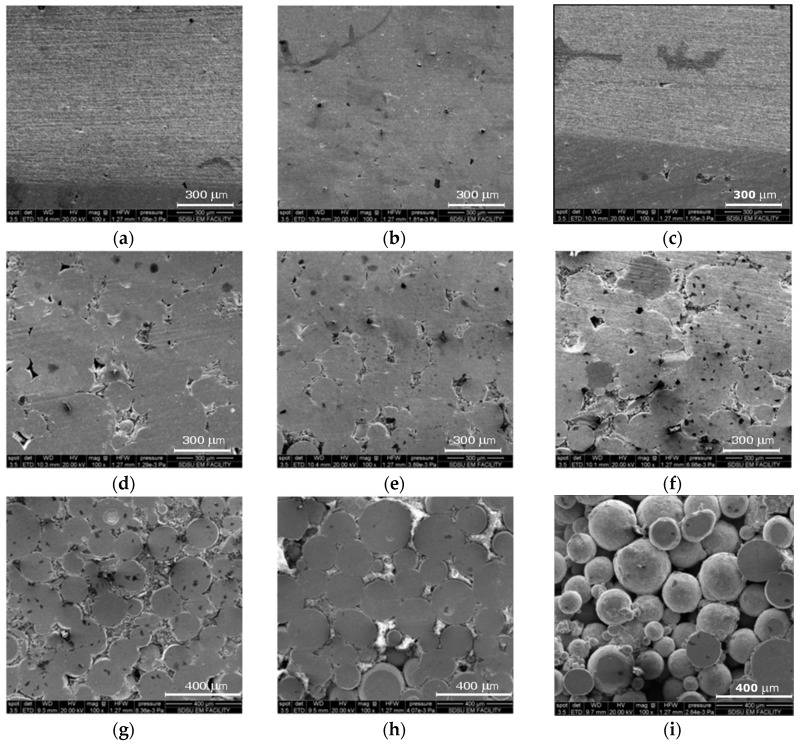
SEM images of selected sintered TNZT samples with varying relative densities. The samples and relative densities of each are as follows: (**a**) 93.8%; (**b**) 89.9%; (**c**) 85.1%; (**d**) 79.1%; (**e**) 73.2%; (**f**) 69.4%; (**g**) 66.0%; (**h**) 59.3% and (**i**) 66.8% (unpolished section).

Figure 9 shows SEM images of selected samples sintered with no holding time. The samples were selected to show the microstructure evolution as a function of porosity.

Study of Figure 9 shows how the spherical powder particles coalesce and merge throughout the sintering process. The spherical nature of the powder is most apparent in Figure 9i, where only a handful of the particles were ground and polished. The circular cross section of these particles is still apparent in some of the other images, but is unrecognizable for all samples achieving a relative density higher than 85%. The dark grey blotches on the surface of the samples are likely to be un-reacted titanium particles.

**Figure 10:**
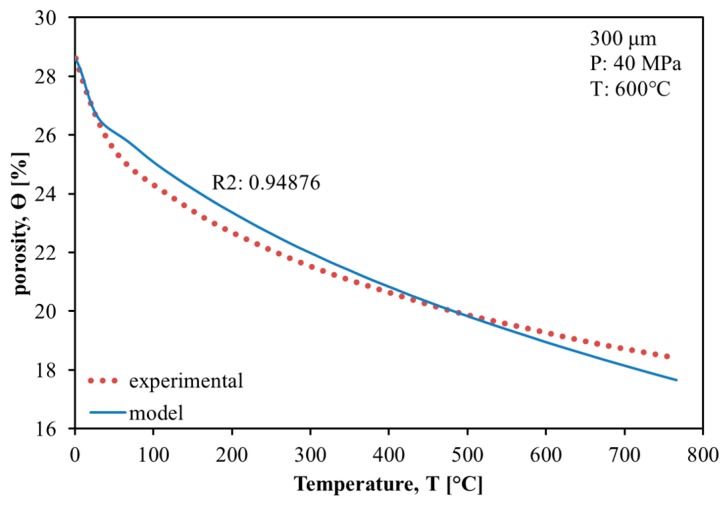
Fit of experimental data (dotted line) using the densification model (result shown with the solid line), R2—correlation coefficient. The results of all the cases analyzed are reported in Table 1.

In Figure 10, an example of the best fit of the experimental data on porosity evolution obtained using the regression analysis is shown (see Section 3):

One can notice that the kinetic model of the porosity evolution corresponds with a good agreement to the experimental data. All the values of activation energy Q obtained from the regression analysis of the experimental data using the kinetic model are reported in Table 1.

In all the cases reported, the minimum error (Equation (3)) obtained comparing experimental and model data it is quite small, where 0.052 is the greatest error value.

(3) err=(∑i=1N(θexp,i−θmod,i)2N)12

The creep activation energy in all the cases analyzed is in good agreement with the value of the creep activation energy for β-Ti present in the literature (153 KJ/mol) [31]. Also, since TNZT is a β alloy, these values of activation energy can be correlated with the DSC curves where any endothermic peak due to the β-transition is not present. Therefore, the obtained results show the reliability and applicability of the proposed densification model, similarly to the previously reported studies [30,38,39,40,41,42]. The modeling results can be used for optimization of the processing of similar powders; they provide a basis for the finite-element analysis of the complex-shape parts′ SPS [28,29,43,44], predicting the evolution of density spatial distribution in the treated specimen’s volume.

## 4. Conclusions

In this study, it was determined that the densification of TNZT was facilitated by higher sintering temperatures, higher pressures, longer dwell times, and smaller initial particles. “Densification maps” were also created. In addition to the densification maps, correlations between the relative density of the TNZT samples and their mechanical properties were developed. It was also determined that all of the samples which were hard enough to assess their strength were sufficiently strong to serve as components of orthopedic implants. 

A densification model is proposed and used to calculate the activation energy based on a regression analysis of the experimental data. Good agreement is found between the experiments and the model results and the model reliability and applicability were confirmed by comparison with the activation energy values of Ti reported in the literature.

## Figures and Tables

**Table 1:** Activation energy (Q) obtained from the regression analysis using Equation (1). Where d is the particle size; P and T are the pressure and temperature of the SPS process, respectively; m is the consolidation parameter; R2 is the correlation coefficient; and err is the minimum error.

d (μm)	P (Mpa)	T (°C)	Q (kJ/mol)	m	R2	Error
300	20	400	153.91	0.23	0.741	0.052
		600	154.00	0.23	0.989	0.001
		800	131.37	0.23	0.987	0.007
	40	400	-	0.23	-	-
		600	153.00	0.23	0.948	0.005
		800	-	0.23	-	--
	60	400	-	0.23	-	-
		600	148.55	0.23	0.997	0.001
		800	-	0.23	-	-
150	20	400	-	0.23	-	-
		600	-	0.23	-	-
		800	145.59	0.23	0.995	0.001
	40	400	-	0.23	-	-
		600	141.65	0.23	0.997	0.001
		800	149.15	0.23	0.949	0.001
	60	400	153.89	0.23	0.824	0.0004
		600	146.23	0.23	0.996	0.001
		800	144.87	0.23	2.699	0.006

## References

[1] Spriggs R.M. (1961). Expression for Effect of Porosity on Elastic Modulus of Polycrystalline Refractory Materials, Particularly Aluminum Oxide. J. Am. Ceram. Soc..

[2] Kawai C., Yamakawa A. (1997). Effect of Porosity and Microstructure on the Strength of Si_3_N_4_: Designed Microstructure for High Strength, High Thermal Shock Resistance, and Facile Machining. J. Am. Ceram. Soc..

[3] Coble R.L., Kingery W.D. (1956). Effect of Porosity on Physical Properties of Sintered Alumina. J. Am. Ceram. Soc..

[4] Knudsen F.P. (1959). Dependence of Mechanical Strength of Brittle Polycrystalline Specimens on Porosity and Grain Size. J. Am. Ceram. Soc..

[5] Robertson D.M., St Pierre L., Chahal R. (1976). Preliminary observations of bone ingrowth into porous materials. J. Biomed. Mater. Res. A.

[6] Head W.C., Bauk D.J., Emerson R.H. (1995). Titanium as the material of choice for cementless femoral components in total hip arthroplasty. Clin. Orthop. Relat. Res..

[7] Bandyopadhyay A., Espana F., Balla V.K., Bose S., Ohgami Y., Davies N.M. (2010). Influence of porosity on mechanical properties and in vivo response of Ti-6Al-4V implants. Acta Biomater..

[8] Cameron H.U., Macnab I., Pilliar R.M. (1978). A porous metal system for joint replacement surgery. Int. J. Artif. Organs.

[9] Evin M.P., Getter L., Adrian J., Cutright D. (1974). Healing of periodontal defects with ceramic implants. J. Clin. Periodontol..

[10] Grandjean-Laquerriere A., Laquerriere P., Guenounou M., Laurent-Maquin D., Phillips T.M. (2005). Importance of the surface area ratio on cytokines production by human monocytes in vitro induced by various hydroxyapatite particles. Biomaterials.

[11] Laquerriere P., Grandjean-Laquerriere A., Jallot E., Balossier G., Frayssinet P., Guenounou M. (2003). Importance of hydroxyapatite particles characteristics on cytokines production by human monocytes in vitro. Biomaterials.

[12] Yablon I.G. (1975). The Effect of Methylmethacrylate on Fracture Healing. Clin. Orthop. Relat. Res..

[13] Revell P.A., Braden M., Freeman M.A.R. (1998). Review of the biological response to a novel bone cement containing poly(ethyl methacrylate) and n-butyl methacrylate. Biomaterials.

[14] El-Warrak A.O., Olmstead M., Schneider R., Meinel L., Bettschart-Wolfisberger R., Akens M.K., Auer J., Von Rechenberg B. (2004). An experimental animal model of aseptic loosening of hip prostheses in sheep to study early biochemical changes at the interface membrane. BMC Musculoskelet. Disord..

[15] El-Warrak A.O., Olmstead M.L., von Rechenberg B., Auer J.A. (2002). A Review of Aseptic Loosening in Total Hip Arthroplasty. Vet. Comp. Orthop. Traumatol..

[16] Taniguchi N., Fujibayashi S., Takemoto M., Sasaki K., Otsuki B., Nakamura T., Matsushita T., Kokubo T., Matsuda S. (2016). Effect of pore size on bone ingrowth into porous titanium implants fabricated by additive manufacturing: An in vivo experiment. Mater. Sci. Eng. C.

[17] Nag S., Banerjee R., Fraser H.L. (2005). Microstructural evolution and strengthening mechanisms in Ti–Nb–Zr–Ta, Ti–Mo–Zr–Fe and Ti–15Mo biocompatible alloys. Mater. Sci. Eng. C.

[18] Özcan M., Hämmerle C. (2012). Titanium as a Reconstruction and Implant Material in Dentistry: Advantages and Pitfalls. Materials.

[19] Niinomi M. (2002). Recent metallic materials for biomedical applications. Metall. Mater. Trans. A.

[20] Hendrickson M., Mantri S.A., Ren Y., Alam T., Soni V., Gwalani B., Styles M., Choudhuri D., Banerjee R. (2017). The evolution of microstructure and microhardness in a biomedical Ti-35Nb-7Zr-5Ta alloy. J. Mater. Sci..

[21] Kuroda D., Niinomi M., Morinaga M., Kato Y., Yashiro T. (1998). Design and mechanical properties of new β type titanium alloys for implant materials. Mater. Sci. Eng. A.

[22] Niinomi M., Kuroda D., Fukunaga K., Morinaga M., Kato Y., Yashiro T., Suzuki A. (1999). Corrosion wear fracture of new β type biomedical titanium alloys. Mater. Sci. Eng. A.

[23] Niinomi M. (1998). Mechanical properties of biomedical titanium alloys. Mater. Sci. Eng. A.

[24] Taddei E.B., Henriques V.A.R., Silva C.R.M., Cairo C.A.A. (2004). Production of new titanium alloy for orthopedic implants. Mater. Sci. Eng. C.

[25] Hutmacher D.W. (2000). Scaffolds in tissue engineering bone and cartilage. Biomaterials.

[26] Ryan G., Pandit A., Apatsidis D.P. (2006). Fabrication methods of porous metals for use in orthopaedic applications. Biomaterials.

[27] Li Y.H., Yang C., Wang F., Zhao H.D., Qu S.G., Li X.Q., Zhang W.W., Li Y.Y. (2015). Biomedical TiNbZrTaSi alloys designed by d-electron alloy design theory. Mater. Des..

[28] Bordia R.K., Kang S.-J.L., Olevsky E.A. (2017). Current understanding and future research directions at the onset of the next century of sintering science and technology. J. Am. Ceram. Soc..

[29] Wei X., Maximenko A.L., Back C., Izhvanov O., Olevsky E.A. (2017). Effects of loading modes on densification efficiency of spark plasma sintering: Sample study of zirconium carbide consolidation. Philos. Mag. Lett..

[30] Bellosi A., Monteverde F., Sciti D. (2006). Fast Densification of Ultra-high-temperature ceramics by spark plasma sintering. Int. J. Appl. Ceram. Technol..

[31] Petersen A.S., Cheung A.M., Neilson H.J., Poon S.J., Shiflet G.J., Lewandowski J.J. (2017). Processing and properties of Ni-based bulk metallic glass via spark plasma sintering of pulverized amorphous ribbons. Mater. Res. Soc. Adv..

[32] Ghasali E., Fazili A., Alizadeh M., Shirvanimoghaddam K., Ebadzadeh T. (2017). Evaluation of Microstructure and Mechanical Properties of Al-TiC Metal Matrix Composite Prepared by Conventional, Microwave and Spark Plasma Sintering Methods. Materials.

[33] Olevsky E.A. (1998). Theory of sintering: From discrete to continuum. Mater. Sci. Eng. R Rep..

[34] Lee G., McKittrick J., Ivanov E., Olevsky E.A. (2016). Densification mechanism and mechanical properties of tungsten powder consolidated by spark plasma sintering. Int. J. Refract. Met. Hard Mater..

[35] Ashby M.F. (1990). HIP 6.0 Background Reading.

[36] Kun W., Zhengyi F., Weimin W., Yucheng W., Jinyong Z., Qingjie Z. (2007). Study on fabrication and mechanism in of porous metals by spark plasma sintering. J. Mater. Sci..

[37] Coble R.L. (1973). Effects of Particle-Size Distribution in Initial-Stage Sintering. J. Am. Ceram. Soc..

[38] Revankar G., Saxena A., Voorhees H., Ritchie R., Shapiro E., Walsh R., Robinson R., Shoberg R.S., Waterhouse R., Roos E. (2000). Harness Testing. Mechanical Testing and Evaluation.

[39] Nakai M., Niinomi M., Akahori T., Tsutsumi H., Ogawa M. (2009). Effect of Oxygen Content on Microstructure and Mechanical Properties of Biomedical Ti-29Nb-13Ta-4.6Zr Alloy under Solutionized and Aged Conditions. Mater. Trans..

[40] Kopova I., Stráský J., Harcuba P., Landa M., Janeček M., Bačákova L. (2016). Newly developed Ti–Nb–Zr–Ta–Si–Fe biomedical beta titanium alloys with increased strength and enhanced biocompatibility. Mater. Sci. Eng. C.

[41] Paneto F.J., Pereira J.L., Lima J.O., Jesus E.J., Silva L.A., Lima E.S., Cabral R.F., Santos C. (2015). Effect of porosity on hardness of Al2O3–Y3Al5O12 ceramic composite. Int. J. Refract. Met. Hard Mater..

[42] Wei X., Back C., Izhvanov O., Haines C.D., Olevsky E.A. (2016). Zirconium carbide produced by spark plasma sintering and hot pressing: Densification kinetics, grain growth, and thermal properties. Materials.

[43] Manière C., Olevsky E.A. (2017). Porosity Dependence of Powder Compaction Constitutive Parameters: Determination Based on Spark Plasma Sintering Tests. Scr. Mater..

[44] Lee G., Olevsky E.A., Manière C., Maximenko A., Izhvanov O., Back C., McKittrick J. (2018). Effect of electric current on densification behavior of conductive ceramic powders consolidated by spark plasma sintering. Acta Mater..

